# Biodegradation of Triphenyl Phosphate by a Novel Marine Bacterium *Pseudomonas abyssi* RL-WG04: Characterization, Metabolic Pathway, Bioremediation and Synergistic Metabolism

**DOI:** 10.3390/toxics14040280

**Published:** 2026-03-26

**Authors:** Min Shi, Danting Xu, John L. Zhou, Yang Jia, Hanqiao Hu, Xingyu Jiang, Yanyan Wang

**Affiliations:** 1 College of Coastal Agricultural Sciences, Guangdong Ocean University, Zhanjiang 524088, China; shim927@stu.gdou.edu.cn (M.S.);; 2 Faculty of Science and Engineering, University of Nottingham Ningbo China, Ningbo 315100, China; john.zhou@nottingham.edu.cn; 3 College of Life and Environmental Science, Wenzhou University, Wenzhou 325035, China; 4 South China Branch of National Saline-Alkali Tolerant Rice Technology Innovation Center, Zhanjiang 524088, China

**Keywords:** microbial remediation, organophosphate, co-culture, bioaugmentation

## Abstract

Triphenyl phosphate (TPHP), a typical organophosphate flame retardant, has been listed as an emerging pollutant, yet its biodegradation remains poorly studied. Herein, an efficient TPHP-degrading marine bacterium, *Pseudomonas abyssi* RL-WG04, was isolated from mangrove sediments, which could degrade 95.22% of 100 mg/L TPHP within 120 h. RL-WG04 exhibited good tolerance to varied environmental conditions, maintaining over 70% TPHP degradation percentages (100 mg/L, 7 d) across 20–50 °C, pH 7.0–9.0, and salinity 2.0–4.0% (NaCl, *w*/*v*). Organic solvents (*p*-xylene, biphenyl, toluene and ethyl acetate, 0.5% *v*/*v*) had a negligible impact, whereas metal ions (Mn^2+^, Fe^3+^, Ca^2+^, Cu^2+^, Mg^2+^, Zn^2+^, and Co^2+^) strongly inhibited degradation, especially at 1 mM. Under optimized conditions, TPHP degradation by RL-WG04 followed the improved Gompertz model (R^2^ = 0.99927). Metabolite identification indicated that RL-WG04 transformed TPHP into phenol but failed to utilize phenol for growth because of the phenol 2-monooxygenase deficiency. Nevertheless, the constructed consortia of RL-WG04 and *Pseudomonas* sp. RL-LY03 (phenol-degrading bacterium) achieved complete TPHP degradation and cell proliferation. Additionally, RL-WG04 could efficiently remove TPHP (25 mg/kg) from clay and sandy mangrove sediments with 100% and 90.04% removal percentages, respectively. Overall, this work provides novel insights into the fate of TPHP and a potential approach for its remediation.

## 1. Introduction

Flame retardants (FRs) are a class of additives that could enhance the flame retardancy and self-extinguishing properties of different materials. To comply with fire safety regulations, the global demand for flame retardants continues to rise. Based on the elemental composition, FRs can be categorized into halogenated FRs, phosphorus FRs, nitrogen FRs, phosphorus–halogen FRs, and phosphorus–nitrogen FRs [1]. Polybrominated diphenyl ethers (PBDEs) are common halogenated FRs. Among them, pentabromodiphenyl ether, octabromodiphenyl oxide, decabromodiphenyl oxide and polychlorinated biphenyls are widely used because of their low cost and excellent flame-retardant properties [2]. However, PBDEs, as persistent organic pollutants (POPs), exhibit long-range transport and bioaccumulation potential [3,4], posing significant risks to the environment and organisms. In 2009, PBDEs were listed under the Stockholm Convention, leading to a global phase-out of traditional halogenated flame retardants [5]. Organophosphate flame retardants (OPFRs), as one of the substitutes for halogenated flame retardants, have been employed as additives in the textile, chemical, electronic, plastic, furniture, and construction industries due to their low cost, variety, and dual functions of flame retardancy and plasticization [6,7,8,9]. They are widely used as flame retardants and defoamers in industrial and household products [10,11,12]. Global OPFR consumption surged from 186,000 tons in 2001 to 680,000 tons in 2015 [13,14], reaching 759,000 tons in 2017, accounting for over 30% of the total flame-retardant market [15]. However, their extensive production and consumption have resulted in frequent detection of OPFRs in air [16], water [17], soil [18], and other environmental matrices. Triphenyl phosphate (TPHP), a typical aromatic OPFR, is widely applied in electronic products, coatings, adhesives, foams, polyvinyl chloride, and resins due to its excellent flame-retardant efficacy [19,20,21,22]. In plastic products, TPHP is frequently employed as an additive to enhance durability and flame retardancy, particularly in PVC products, food packaging materials, and children’s toys [23,24]. In the realm of consumer goods, it is also widely applied as a plasticizer in personal care items such as nail polish to increase the toughness and durability of the coating [25]. TPHP is typically incorporated into products as a chemical additive through physical methods, making it prone to environmental release via leaching. Its persistence and resistance to degradation facilitate bioaccumulation through the food chain, posing ecological and health risks. TPHP has been detected in air [26,27], seawater [28], sediments [27], and biota [29,30,31]. For instance, Pantelaki et al. analyzed the concentration of OPFRs in coastal waters of Thessaloniki, Greece, and revealed that the dissolved TPHP concentrations ranged from 400 ng/L to 1270 ng/L with a 100% detection rate [32]. Ding et al. investigated OPFRs bioaccumulation in a tropical marine food web in the South China Sea, detecting TPHP in phytoplankton, zooplankton, oysters, crabs, and fish [33]. Additionally, TPHP was found at concentrations as high as 350 ng/g (lipid weight) in adult coral groupers from Manila Bay, Philippines [34]. TPHP is widely accumulated in organisms and poses a potential threat to the health of organisms.

Environmental exposure is the primary source of TPHP accumulation in organisms, mainly through inhalation, ingestion, and dermal contact. Studies have demonstrated that TPHP exhibits neurotoxicity [35], reproductive toxicity [36,37,38], developmental toxicity [39], endocrine-disrupting effects [40], mutagenicity, and carcinogenicity [41]. Liu et al. investigated the toxic mechanism of TPHP to *Phaeodactylum tricornutum* and revealed that TPHP induces reactive oxygen species (ROS) accumulation and disrupts lipid biosynthesis, leading to subcellular structural damage [42]. Aslantürk et al. evaluated the genotoxicity and cytotoxicity of TPHP on onion root tip cells and found that TPHP exposure reduced the mitotic index and increased chromosomal aberration rates, which might serve as indicators of enhanced cytotoxicity and genotoxicity [38]. Wang et al. determined the TPHP-induced hepatotoxicity pathways, and the results demonstrated that TPHP triggers apoptosis in human hepatocytes, disrupts cellular ultrastructure, and elevates ROS levels, indicating significant impacts on apoptosis, oncogene activation, redox homeostasis, and DNA damage repair processes [27]. The Safety Data Sheet from Sigma-Aldrich/Merck (Version: 10.2, Issue Date: 10 December 2025) explicitly states that TPHP is highly toxic to multiple aquatic organisms (including *Oncorhynchus mykiss*, *Daphnia magna*, *Desmodesmus subspicatus*, *Pimephales promelas* and *Daphnia magna*) and may cause long-term adverse effects. Furthermore, it has been reported that TPHP is particularly toxic to aquatic ecosystems. Under TPHP stress, microalgae exhibited a significant decrease in growth rate, accompanied by notable alterations in metabolites. The underlying toxic mechanism involves disruption of cell membrane integrity and a potential downregulation of ROS (reactive oxygen species) levels [43]. Shi et al. explored the molecular mechanisms underlying the acute developmental toxicity of TPHP in zebrafish larvae, and the results demonstrated that TPHP exposure significantly interfered with the expression of key visual proteins, resulting in abnormal eye development and functional impairment, thereby affecting survival and population sustainability [44]. Given its toxicity and bioaccumulative potential, TPHP has emerged as a concerning environmental pollutant.

On 7 November 2024, the European Chemicals Agency officially listed TPHP as a Substance of Very High Concern candidate (https://www.echa.europa.eu/web/guest/registry-of-svhc-intentions/-/dislist/details, accessed on 3 January 2026). Consequently, efficient and environmentally friendly methods for the elimination of TPHP from the environment are of great concern. Current TPHP removal methods primarily include physical adsorption [45], photocatalysis [46], chemical oxidation [47], and biodegradation [48]. Although chemical and physical methods can effectively degrade TPHP, biodegradation offers advantages such as high efficiency, stability, and minimal secondary pollution. Wei et al. isolated *Brevibacillus brevis* from an e-waste dismantling area, which achieved a degradation efficiency of 92.1% for 3 μmol/L TPHP within 5 d at pH 7.0 and 30 °C [49]. Similarly, Kawagoshi et al. isolated *Roseobacter* sp. YS-57, which completely degraded 25 mg/L TPHP within 14 h [48]. Wang et al. reported that *Sphingopyxis* sp. GY-2 degraded 100 mg/L TPHP with 99.8% efficiency within 4 d [50]. Long et al. demonstrated that *Rhodococcus pyridinivorans* YC-MTN degraded 99.2% of 100 mg/L TPHP within 3 d [51]. Additionally, strains YC-SY1, YC-BJ1, and YC-GZ1 degraded 79.4%, 99.8%, and 99.6% of 100 mg/L TPHP within 4 d, respectively [52,53,54]. Nevertheless, although the detection of TPHP in marine ecosystems has been widely reported, there are relatively few studies on the biodegradation of TPHP, particularly concerning TPHP-degrading bacteria isolated from marine environments. Therefore, the discovery of novel marine TPHP-degrading microbes is crucial for enriching microbial degradation resources and understanding the fate of OPFRs in the marine ecosystem.

In this study, a novel marine bacterial strain, *Pseudomonas abyssi* RL-WG04, capable of efficiently degrading TPHP, was isolated from mangrove sediments in the intertidal zone of Zhanjiang Bay, China. The degradation characteristics of the strain RL-WG04, the metabolic pathway of TPHP, and its application potential in the bioremediation of TPHP-contaminated mangrove sediments were investigated. Genomic functional analysis of the strain RL-WG04 was conducted, and the synergistic degradation effects on the complete mineralization and utilization of TPHP were explored. These findings provide theoretical support for expanding the diversity of TPHP-degrading microorganisms, elucidating the transformation mechanisms of OPFRs in marine environments, and advancing bioremediation strategies to tackle environmental pollution.

## 2. Materials and Methods

### 2.1. Chemicals and Medium

TPHP (AR, 99.0%), toluene (AR, 99.5%), and ethyl acetate (AR, 99.5%) were purchased from Sinopharm Chemical Reagent Co., Ltd. (Shanghai, China). Phenol (AR, 99.0%), methanol (HPLC, 99.9%), *n*-hexane (HPLC, 99.9%), and acetonitrile (HPLC, 99.9%) were purchased from Macklin Biochemical Co., Ltd. (Shanghai, China). Other common analytical-grade chemicals, such as *p*-xylene (AR, 99.0%), NaCl, KCl, and CaCl_2_, were purchased from Guangzhou Chemical Reagent Factory (Guangzhou, China). The biological kits and enzymes used in this study were purchased from Takara Biomedical Technology (Beijing, China).

Luria–Bertani (LB) medium was used for the enrichment of the TPHP-degrading bacterium, while minimal salt medium (MSM) was applied for the isolation of the TPHP-degrading bacterium. The composition of these two media was as follows:

LB medium: NaCl 10 g/L, tryptone 10 g/L, and yeast extract 5 g/L.

MSM: NaCl 10 g/L, (NH_4_)_2_SO_4_ 2 g/L, MgSO_4_·7H_2_O 0.2 g/L, Na_2_HPO_4_·12H_2_O 1.5 g/L, KH_2_PO_4_ 1.5 g/L, KNO_3_ 1 g/L, and CaCl_2_ 0.01 g/L.

The solid media of LB and MSM were prepared by supplementing agar with a final concentration of 15 g/L. The pH of the media was adjusted to 7.0 ± 0.2, and the media was sterilized by autoclaving (121 °C, 30 min) before being stored for subsequent use.

### 2.2. Sample Collection

The sediment samples for the isolation of TPHP-degrading and phenol-degrading bacteria were collected from the intertidal mangrove area (21°209′683″ N, 110°421′363″ E) of Zhanjiang Bay, China. The sandy sediment samples used for the bioremediation experiment were collected from the same intertidal mangrove area (21°209′683″ N, 110°421′363″ E), while the clay sediment was collected from the mangrove area of Techeng Island (21°147′942″ N, 110°442′261″ E) of Zhanjiang, China. The physicochemical characteristics of clay and sandy sediments are presented in Supplementary Material Table S1. During the sediment collection, the surface sediment layer (3 cm to 5 cm depth) was removed, and subsurface soil (5 cm to 10 cm depth) was excavated. Following the removal of rocks and plant residues, bacterial isolation and enrichment were performed.

### 2.3. Isolation and Identification of TPHP-Degrading Strain

TPHP-degrading bacteria were enriched and domesticated using the sole carbon source method. The specific steps were as follows: (a) First, 1 g of mangrove sediment was added into 100 mL of MSM containing 100 mg/L TPHP and incubated in a shaker at 30 °C and 180 rpm in the dark for 7 d to isolate TPHP-degrading bacteria. (b) Then, 10 mL of culture was transferred into 90 mL of MSM containing 200 mg/L TPHP and incubated (30 °C and 180 rpm) in the dark for another 7 d. (c) This process was repeated until the concentration of TPHP was increased to 500 mg/L. (d) The culture was streaked onto a solid MSM containing 100 mg/L TPHP and incubated in a constant temperature incubator at 30 °C for 5 d in the dark. (e) A single colony with a hydrolysis halo was selected and inoculated into 10 mL of liquid LB medium, followed by incubating in a shaker at 30 °C and 180 rpm for 1 d in darkness to obtain an OD_600_ of ~1.0. (f) Bacterial cells were harvested by centrifuging 1 mL of LB culture, washed three times with phosphate-buffered saline (PBS, 0.1 M, pH 7.4), resuspended in 1 mL MSM, and inoculated into MSM liquid medium containing 100 mg/L TPHP. Cultures were incubated at 30 °C with 180 rpm in the dark for 5 d. (g) The residual concentration of TPHP in the culture was determined by liquid chromatography. Isolates with a degradation percentage exceeding 90% were selected and subjected to steps (d)–(g) at least three times to ensure that a pure and stable TPHP-degrading bacterial strain was isolated. Phenol-degrading bacteria were also isolated using the sole carbon source method, with specific procedures outlined in Supplementary Material Text S1.

The isolated TPHP-degrading bacterial strains were identified via physiological and biochemical characteristics analysis, phylogenetic analysis based on the 16S rRNA gene, and genome-based taxonomy. The physiological and biochemical characteristics of isolated strains were analyzed according to the Handbook for Systematic Identification of Common Bacteria [55]. Scanning electron microscopy was used to observe the cellular morphology of the target strain at the microscopic level. Genomic DNA was extracted using the TaKaRa MiniBEST Bacterial Genomic DNA Extraction Kit (Takara, Beijing, China), and 16S rRNA gene amplification was performed with universal primers 27F/1492R. The PCR products were inserted into pMD19-T vectors (Takara, Beijing, China) and sequenced by Suzhou Genewiz Biotechnology Co., Ltd. Sequencing results were analyzed using DNASTAR Lasergene software (version 11.1.0.54), with manual corrections to remove vector sequences, yielding complete 16S rRNA sequences of the target strain. Genus-level identification was performed using BLAST (version 2.17.0) (https://blast.ncbi.nlm.nih.gov/Blast.cgi, accessed on 3 January 2026) alignment, and reference strains were retrieved from the List of Prokaryotic Names with Standing in Nomenclature (https://lpsn.dsmz.de). Phylogenetic analysis was conducted using MEGA 11.0 software, with a phylogenetic tree constructed by the neighbor-joining method (bootstrap value = 1000).

The complete genome sequencing of the TPHP-degrading bacterium was conducted by Biomarker Technologies Corporation (Beijing, China) using a PacBio HiFi platform according to the standard protocol provided by Pacific Biosciences (Menlo Park, CA, USA). After library sequencing, the CCS reads with excessively short lengths were filtered out. Hifiasm was used to assemble the filtered CCS reads; subsequently, Circlator v1.5.5 was employed to circularize and adjust the starting site, while Pilon v1.22 was used to correct the assembled genome using second-generation sequencing data, resulting in a more accurate genome for further analysis. Gene functional annotation encompassed general databases such as the Non-Redundant Protein Database (Nr), Gene Ontology (GO), and Kyoto Encyclopedia of Genes and Genomes (KEGG), as well as other databases. The genome was visualized using Circos v0.69. Through the Rapid Annotation using Subsystem Technology (RAST) data analysis platform (https://rast.nmpdr.org), the genome information of the strain was compared with that of previously reported strains, and related metabolic pathways were annotated in the genome of the TPHP-degrading strain.

According to the results of 16S rRNA gene sequencing and whole-genome sequencing of the TPHP-degrading bacteria strain, the genomes of strains closely related to the TPHP-degrading bacterium were subjected to average nucleotide identity (ANI) calculation. The ANI calculator (https://www.ezbiocloud.net/tools/ani, accessed on 3 January 2026) from the EzBioCloud database was employed to calculate ANI values between the strains and the TPHP-degrading bacterium, followed by result analysis. The identification of phenol-degrading bacteria primarily relies on 16S rRNA gene analysis, which aligns with the 16S rRNA-based characterization of the aforementioned TPHP-degrading bacteria.

### 2.4. Degradation Characteristics of TPHP-Degrading Strain

#### 2.4.1. Effects of Different Environmental Factors on Degradation Ability

The single colony of the TPHP-degrading bacterium on the LB plate was selected, inoculated into 10 mL LB liquid medium, and incubated under constant shaking (30 °C, 180 rpm) for 1 d. Bacterial cells were harvested by centrifugation at 5000× *g* for 5 min, washed three times with PBS (0.1 M, pH 7.4), and finally resuspended in fresh MSM liquid medium with a final cell density of OD_600_ = 1.0. The cell suspension served as the inoculant, and the inoculation ratio was maintained at 1.0% (*v*/*v*).

To determine the half-maximal effective concentration (EC_20_ and EC_50_) of TPHP against strain RL-WG04, the inhibitory effects of varying TPHP concentrations on its growth were quantitatively assessed. In the experiment, 0.2% (*v*/*v*) glucose was added to the MSM as a carbon source to ensure uniform nutritional conditions across all treatment groups. Subsequently, TPHP was supplemented at concentrations of 0, 25, 50, 100, 200, 300, 400, 500, 600, and 700 mg/L. After incubation in the dark at 30 °C and 180 rpm for 24 h, the samples were harvested by centrifugation at 5000× *g*. The supernatant was discarded, and the cell pellet was resuspended in PBS (the wash and centrifugation were repeated three times). Finally, the cells were resuspended in buffer, and the OD_600_ value was measured. All treatments were performed in triplicate. The inhibition rate was calculated using Equation (1) [56].

Calculation formula for inhibition rate:

(1) $$Inhibition\;rate\;(\%)=(1-\frac{{OD}_{c}}{{OD}_{0}})*100\%$$

where *OD_c_* represents the OD_600_ value after 24 h of incubation at a specific TPHP concentration, and *OD*_0_ represents the cell density in the absence of TPHP (at 0 mg/L).

The effects of different environmental factors on the TPHP (100 mg/L) degradation ability of the isolated strain were determined via single-factor assay, including temperature (10 °C, 20 °C, 30 °C, 40 °C, and 50 °C), initial pH (3, 4, 5, 6, 7, 8, 9, 10, and 11), and salinity (NaCl, 2%, 4%, 6%, 8%, 10%, and 12% *w*/*v*). The degradation percentage of TPHP was calculated after 7 d of incubation at 180 rpm under different environmental conditions. In these experiments, the MSM containing TPHP (100 mg/L) without inoculation served as the control treatment. All experiments were conducted in triplicate.

Glycogen, glucose-6-phosphate, citric acid, succinic acid, acetic acid, L-glutamic acid, L-alaninamide, and putrescine were individually added to the MSM as the sole carbon or nitrogen source. The OD_600_ value was measured after 7 days of cultivation in the dark at 30 °C and 180 rpm. Uninoculated MSM served as the control treatment. All experiments were performed in triplicate.

#### 2.4.2. Degradation Kinetics of TPHP by Target Strain

The first-order decay model (Equation (1)) and the improved Gompertz model (Equation (2)) were employed to simulate the degradation kinetics of TPHP (100 mg/L) under the optimized conditions [57,58].

The first-order decay model:

(2) $$y=A*exp\left(-\frac{t}{t_{1}}\right)+y_{0}$$

The improved Gompertz model:

(3) $$y=y_{0}-A*exp\left(-exp\left(\frac{V_{m}*e}{A}\right)*\left(L-t\right)+1\right)$$

where *y* represents the substrate concentration, *y*_0_ represents the initial concentration, *A* represents the biodegradation potential, *V_m_* represents the maximum biodegradation rate, and *L* is the lag phase.

#### 2.4.3. Tolerance of the Strain to Organic Solvents and Metal Ions

To explore the effects of organic solvents and metal ions on the TPHP degradation capability of the isolated strain, a bacterial suspension was prepared as mentioned above and inoculated into MSM liquid medium containing 100 mg/L TPHP. Following the single-variable principle, 18 treatment groups were established: (1) four common organic solvents (*p*-xylene, biphenyl, toluene, and ethyl acetate, 0.5% *v*/*v*) and (2) seven metal ions (Mn^2+^, Fe^3+^, Ca^2+^, Cu^2+^, Mg^2+^, Zn^2+^, and Co^2+^) with two concentrations (0.5 and 1 mM; a total of 14 treatments). The MSM liquid medium (containing 100 mg/L of TPHP) with inoculation but without supplementation of the above organic solvents and metal ions was used as the control treatment, while the MSM liquid medium (100 mg/L of TPHP, containing the above-mentioned organic solvents or metal ions) without inoculation was applied as the abiotic control. All cultures were incubated under constant shaking at 30 °C and 180 rpm in the dark for 5 d, and the residual concentration of TPHP was determined thereafter. All treatments were conducted with three replicates.

### 2.5. Analysis of Metabolic Pathway

To gain an insight into the metabolic mechanism of TPHP by the isolated strain, the metabolic pathway was inferred by identifying the metabolic intermediates. The experimental procedure was as follows: (a) The bacterial suspension was prepared according to the aforementioned method, and 1.0% (*v*/*v*) of the suspension was inoculated into MSM liquid medium containing 100 mg/L TPHP. (b) Cultures were incubated in a rotary shaker (30 °C and 180 rpm) in the dark, and samples were collected on the 2nd and 3rd days. (c) An equal volume of *n*-hexane was added into the culture, followed by ultrasonic extraction (30 kHz, 150 W) for 10 min. (d) The organic phase was transferred into an empty flask, and the pH of the aqueous phase was adjusted to 5.0 for the extraction. (e) The organic phases were merged, and the organic solvents were evaporated using a rotary evaporator. (f) The extracts were redissolved in methanol, filtered through a 0.22 μm organic phase filter membrane, and analyzed using a Thermo Vanquish high-performance liquid chromatography–mass spectrometry (HPLC-MS, Thermo Fisher Scientific, Waltham, MA, USA).

### 2.6. Co-Culture of TPHP-Degrading Bacteria and Phenol-Degrading Bacteria

With the aim of determining whether phenol-degrading bacteria can co-degrade TPHP with TPHP-degrading bacteria and utilize TPHP metabolites to promote bacterial growth, a co-culture experiment involving a phenol-degrading bacterium and a TPHP-degrading bacterium was conducted. The isolation and identification of a phenol-degrading bacterial strain are presented in Supplementary Material Text S1. The following assays aimed to assess the growth of the strains during the degradation of the target substrate (phenol or TPHP), as well as the degrading capability of phenol or TPHP. The experimental design is shown in Table 1, and all assays were carried out with 10 mL of MSM containing 100 mg/L of target substrate as the sole carbon source. Briefly, treatments (1) and (2) were used to determine the TPHP-degrading capability of TPHP-degrading and phenol-degrading strains, treatments (3) and (4) were applied for determining the phenol-degrading capability of TPHP-degrading and phenol-degrading strains, and treatment (5) was employed for measuring the synergistic degradation of TPHP by TPHP-degrading and phenol-degrading strains. The same cultures (containing the same concentration of target substrate) without inoculation were set as an abiotic control. All treatments were incubated under constant shaking (30 °C, 180 rpm), and each treatment was conducted in three replicates. The concentrations of TPHP and phenol were determined every 24 h. Throughout the 7-day cultivation period, the cell density (OD_600_) of each treatment group was measured every 12 h. The growth curve of treatment (5) was fitted using the sigmoidal Richards model (Equation (4)).

The sigmoidal Richards model:

(4) $$y={\left(y_{max}^{1-d}+exp\left(-k*\left(x-x_{c}\right)\right)\right)}^{\frac{1}{1-d}}$$

where *y_max_* is the maximum cell density, *d* is the shape parameter, *k* is the growth rate parameter, and *x_c_* is the center offset.

### 2.7. Bioremediation of TPHP-Contaminated Mangrove Sediments

To evaluate the application potential of an isolated TPHP-degrading bacterial strain, mangrove sediment samples were collected from the intertidal mangrove area for the bioremediation assays. These samples were used to assess the strain’s TPHP removal efficiency in two kinds of mangrove sediment: common clay sediment and loose sandy sediment. Each sediment type was divided into two groups: one subjected to autoclave sterilization (121 °C for 30 min) and the other left untreated. A total of four treatments were prepared, each containing 40 g of sediment: (i) sterilized clay sediment, (ii) non-sterilized clay sediment, (iii) sterilized sandy sediment, and (iv) non-sterilized sandy sediment. For each treatment, TPHP was added with a final concentration of 25 mg/kg, and 1 mL of TPHP-degrading bacterial suspension was inoculated. After thorough mixing, the samples were incubated in an artificial climate incubator. The incubation conditions were set as follows: temperature at 30 °C, relative humidity of 80%, and a light period of 14 h and a dark period of 10 h. Residual concentration of TPHP was analyzed by high-performance liquid chromatography (HPLC) every 2 d.

### 2.8. Analytic Methods

In this study, ultrasonic extraction was employed to extract TPHP from the liquid medium. The specific procedures were as follows: An equal volume of *n*-hexane was added to the sample and subjected to ultrasonic-assisted extraction (40 kHz, 10 min). The extract was collected, concentrated using a rotary evaporator, and finally reconstituted in methanol. For sediment samples, TPHP was extracted with *n*-hexane in three successive extractions. After combining the extraction solutions, the mixture was concentrated by rotary evaporation and redissolved in methanol. The extract was filtered through a 0.22 μm membrane filter, and the TPHP content in the sample was determined using an Agilent 1260 series HPLC system, in strict accordance with the experimental protocol. The detection conditions were as follows: an Eclipse XDB-C18 column (4.6 × 250 mm, 5 μm); a mobile phase consisting of 80% methanol and 20% water at a flow rate of 1 mL/min; an injection volume of 5 μL; column temperature maintained at 28 °C; and detection wavelength set at 250 nm. The retention time for TPHP was 5 min. A calibration curve relating TPHP peak area to concentration was generated using Origin 2024 software (Figure S1). The removal percentage of TPHP was calculated using the following formula:

(5) $$Removal\;percentage\;(\%)=\frac{C_{n}-C_{t}}{C_{n}}*100\%$$

where *C_n_* represents the substrate concentration in the control group at time *t*, and *C_t_* denotes the substrate concentration in the experimental group at time *t*.

Metabolite analysis was performed using HPLC-MS (Thermo Fisher Scientific, Waltham, MA, USA). The system was equipped with a Thermo Q Exactive Focus mass spectrometer featuring an electrospray ionization source (ESI). The spray voltage was set to 3.50 kV in positive ion mode and -2.50 kV in negative ion mode. Data were acquired in both ESI positive and negative ionization modes simultaneously during sample analysis. The capillary temperature was maintained at 325 °C. The mass resolution was set to 70,000 for the full scan and 17,500 for the MS/MS scan. Chromatographic separation was achieved using an ACQUITY UPLC^®^ HSS T3 column (2.1 × 150 mm, 1.8 μm; Waters, Milford, MA, USA). The flow rate was 0.25 mL/min, the column temperature was 40 °C, and the injection volume was 2 μL. The mobile phase for positive ion mode consisted of acetonitrile with 0.1% formic acid and water with 0.1% formic acid. For negative ion mode, the mobile phase comprised acetonitrile and aqueous ammonium formate (5 mM).

An ultraviolet–visible spectrophotometer (model P4PC, MAPADA, Shanghai, China) was used to measure the absorbance of the bacterial culture at 600 nm to monitor bacterial growth.

### 2.9. Statistical Methods

Statistical analysis was conducted using SPSS version 24.0 (IBM, Armonk, NY, USA). Data are expressed as mean ± standard error, with a statistical significance level set at *p* < 0.05. All statistical results in this study are based on the average of at least three independent replicates.

### 2.10. Data Availability

The 16S rRNA gene sequence and whole-genome sequence of strain RL-WG04 have been deposited in GenBank under accession numbers PV770161 and PRJNA1311828, respectively.

## 3. Results

### 3.1. Identification of TPHP-Degrading Strain

**Figure 1:**
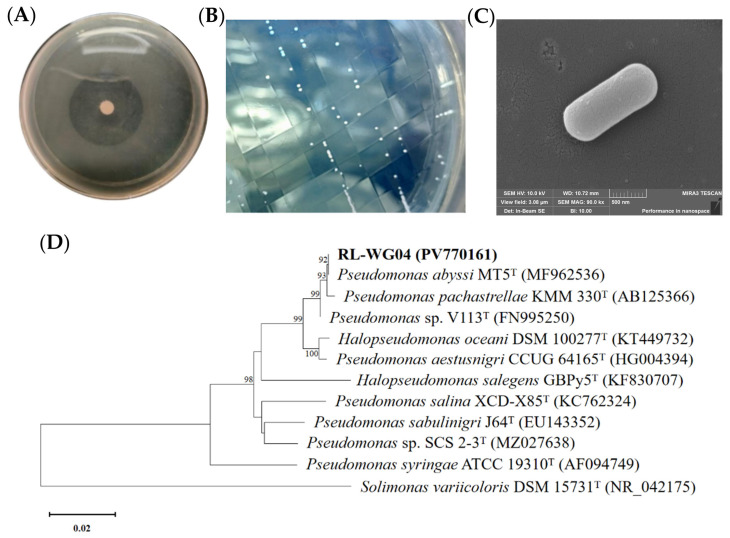
Identification of strain RL-WG04. (**A**) Hydrolysis of TPHP by strain RL-WG04 on MSM plate; (**B**) colony morphology of strain RL-WG04 on LB plate; (**C**) cell morphology of strain RL-WG04; (**D**) phylogenetic analysis based on 16S rRNA gene. T, type strain.

After repeated enrichment and purification several times, a bacterial strain capable of efficiently degrading TPHP was isolated from mangrove sediments and designated as RL-WG04. Hydrolysis of TPHP on the MSM plate by RL-WG04 resulted in the formation of a transparent hydrolysis halo (Figure 1A), while the colony of strain RL-WG04 on the LB plate was smooth with flat edges, forming an opaque, milky-white circular colony (Figure 1B). The cells of strain RL-WG04 were short, rod-shaped, and lacked pili and flagella (Figure 1C). RL-WG04 was positive for Gram staining, the catalase test, and gelatin hydrolysis but negative for starch hydrolysis, indole production, the methyl red test, and the Voges–Proskauer (V–P) test. It was resistant to kanamycin but susceptible to ampicillin and tetracycline. The alignment of the 16S rRNA gene with known records indicated that strain RL-WG04 belongs to the genus *Pseudomonas*. Phylogenetic analysis (Figure 1D) revealed that strain RL-WG04 was clustered with *Pseudomonas abyssi* MT5^T^, and the bootstrap value was 92%. Further, ANI calculation indicated that the ANI value between RL-WG04 and *Pseudomonas abyssi* MT5^T^ was the highest, reaching 98.38% (Table 2). Given that ANI values higher than 96% are indicative of the same species [59], strain RL-WG04 and *Pseudomonas abyssi* MT5^T^ were classified as the same species. Based on the physio-biochemical characterization, phylogenetic analysis, and ANI calculation, strain RL-WG04 was identified as *Pseudomonas abyssi*. To our knowledge, this represents the first report of a *Pseudomonas* species capable of degrading TPHP.

### 3.2. Characterization of Strain RL-WG04

**Figure 2:**
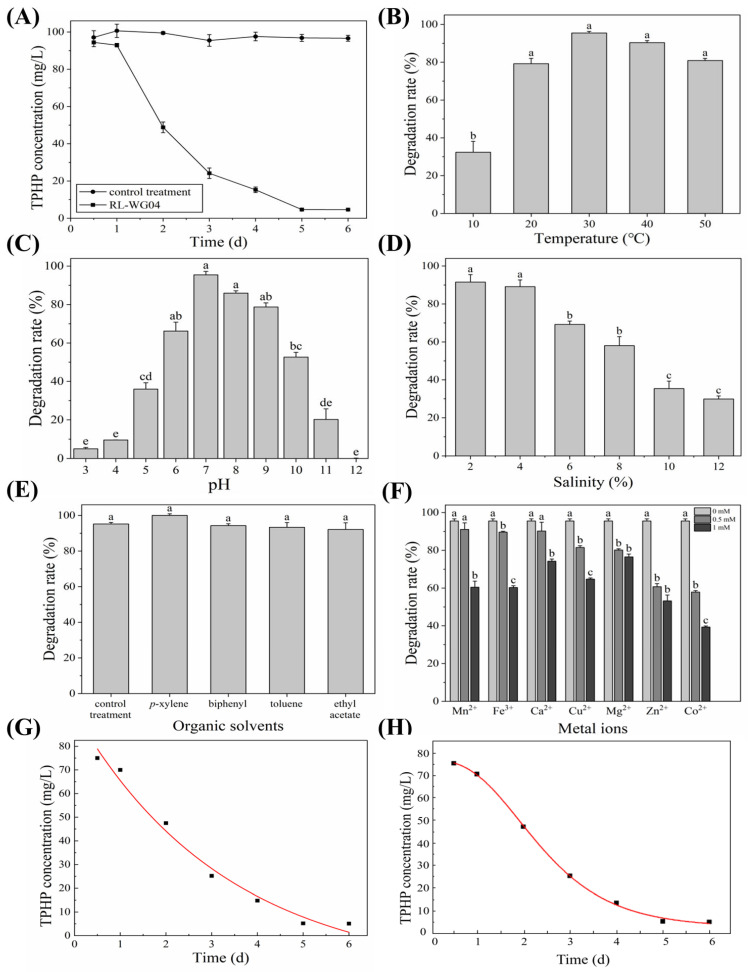
Characterization of strain RL-WG04. (**A**) Degradation of TPHP; (**B**) effects of temperature; (**C**) effects of pH; (**D**) effects of salinity; (**E**) effects of organic solvents; (**F**) effects of metal ions; (**G**) the fitted curve for TPHP degradation with the first-order kinetic model; (**H**) the fitted curve for TPHP degradation with the improved Gompertz model. Different lowercase letters indicate statistically significant differences between groups (*p* < 0.05).

Generally, the application potential of a specific strain was influenced by its environmental adaptability. In this study, the effects of carbon/nitrogen sources and TPHP concentration on the growth of strain RL-WG04 were evaluated. Additionally, the impacts of initial pH, temperature, salinity, organic solvents, and metal ions on the degradation of TPHP by the strain were investigated, and the optimal conditions for TPHP degradation were determined. In the growth inhibition assay, in the absence of TPHP (0 mg/L), the OD_600_ value of strain RL-WG04 was 1.489, corresponding to an inhibition rate of 0%. When the TPHP concentration was 100 mg/L, the inhibition rate only increased to 14.80%, indicating that the inhibitory effect on the microorganism was very mild and scarcely affected normal physiological activities (Figure S2). The TPHP concentrations corresponding to EC_20_ and EC_50_ were approximately 123 mg/L and 270 mg/L, respectively. In the experiment using a single compound as the sole carbon or nitrogen source, strain RL-WG04 was able to grow on glycogen, citric acid, and L-glutamic acid but could not utilize glucose-6-phosphate, succinic acid, acetic acid, L-alaninamide, or putrescine. As shown in Figure 2A, the concentration of TPHP decreased gradually from 12 h to 24 h, with a degradation percentage of 7.05% at 24 h, indicating that the strain exhibited a lag phase in the initial stage of degradation. From 24 h to 120 h, TPHP concentration declined rapidly, reaching a degradation percentage of 95.22% at 120 h. As shown in Figure 2B, the degradation percentage of 100 mg/L TPHP by strain RL-WG04 in 120 h exceeded 75% at temperatures ranging from 20 to 50 °C, with the maximum degradation percentage (95.53%) achieved at 30 °C. This indicated that strain RL-WG04 exhibits strong adaptability to a wide range of temperatures. Strain RL-WG04 also showed good tolerance to different initial pH (Figure 2C). The degradation percentages of TPHP were 35.97%, 66.12%, 95.47%, 85.92%, 78.80%, and 52.63% for pH 5.0, 6.0, 7.0, 8.0, 9.0, and 10.0, respectively. These results indicate that strain RL-WG04 achieved the highest TPHP degradation percentage (95.47%) at pH 7.0. As expected, strain RL-WG04 exhibited good tolerance to salinity and degradation capability under high salt concentrations (Figure 2D). Although the degradation percentage of TPHP decreased with the increase in salinity, strain RL-WG04 could degrade 89.08% of 100 mg/L TPHP in 120 h under a salinity of 4.0%. Meanwhile, approximately 57.99% of TPHP (100 mg/L) was degraded by strain RL-WG04 in 120 h under a salinity of 8.0%. As shown in Figure 2E, the degradation percentages of TPHP (100 mg/L) exceeded 90% with the addition of 0.5% (*v*/*v*) of four organic solvents in 120 h. This suggests that low concentrations of organic solvents had limited impacts on the degradation of TPHP by strain RL-WG04. Additionally, seven metal ions reduced the degradation percentage of TPHP, with higher concentrations exerting more pronounced inhibitory effects (Figure 2F). At the concentration of 1 mM, all seven metal ions significantly inhibited the degradation of TPHP, with Co^2+^ reducing the degradation percentage of TPHP to 39.38% (100 mg/L, 120 h). Finally, under optimized conditions (30 °C and an initial pH of 7.0), the degradation of TPHP was measured, and the degradation kinetics were analyzed. Further analysis of the degradation kinetics of TPHP indicated that the improved Gompertz model exhibited greater applicability than the first-order decay model. As shown in Figure 2G, H, the degradation process of TPHP by strain RL-WG04 could be well described by the improved Gompertz model. The fitting results of this model (Equation (6)) yielded a high correlation coefficient (R^2^ = 0.99927), while those of the first-order decay model (Equation (7)) produced an R^2^ value of 0.97731. The degradation kinetics analysis revealed a biodegradation potential range of 74.67 mg/kg, a maximum biodegradation rate of 25.80 mg/kg/d, and a lag period of 0.84 d.

The improved Gompertz model:

(6) $$y=77.37-74.67*exp\left(exp\left(\frac{25.80*2.71}{74.67}*\left(0.84-t\right)+1\right)\right)$$

The first-order decay model:

(7) $$y=111.35*exp\left(-\frac{t}{3.33}\right)-16.94$$

### 3.3. Metabolic Intermediates and Metabolic Mechanism of TPHP

**Figure 3:**
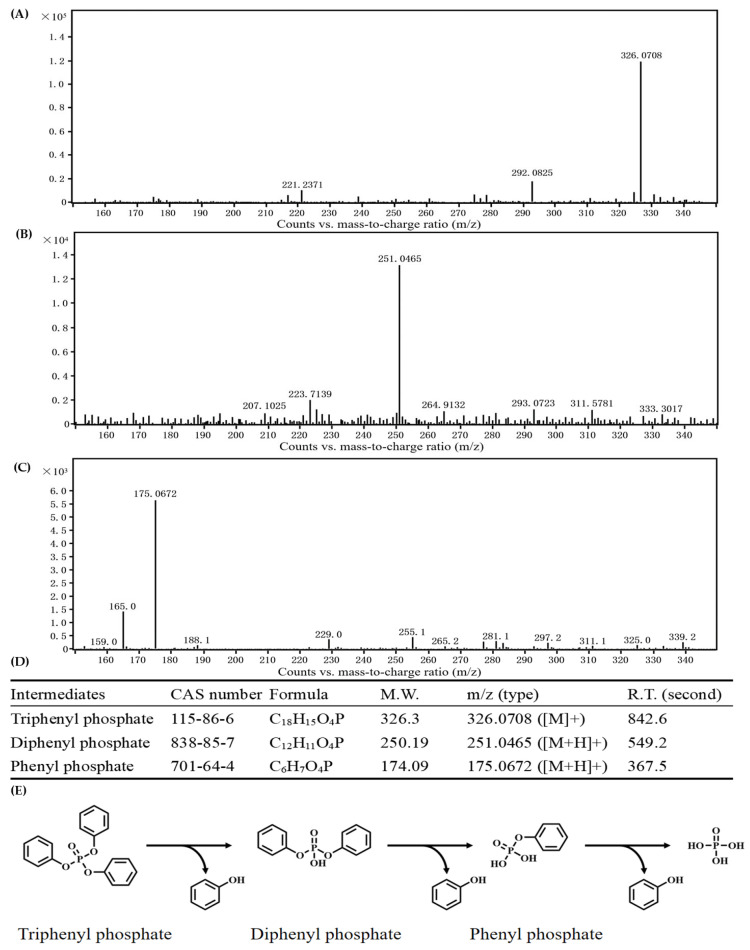
Identification of TPHP metabolic intermediates (**A**–**D**) and deduction of its metabolic pathway in strain RL-WG04 (**E**).

To elucidate the metabolic mechanism of TPHP in strain RL-WG04, the metabolites of TPHP during the degradation were analyzed, and the metabolic pathway was deduced thereafter. During TPHP biodegradation, except for the parent compounds (Figure 3A), two major metabolic intermediates of TPHP were identified, viz. diphenyl phosphate (Figure 3B) and phenyl phosphate (Figure 3C). Although diphenyl phosphate and phenyl phosphate could not be detected on the 5th day, phenol was found to be accumulated in culture, and the molar concentration of phenol was approximately three times the initial TPHP concentration (Figure S3). The identification of metabolic intermediates is summarized in Figure 3D. These results suggest that TPHP was fully transformed into phenol via diphenyl phosphate and phenyl phosphate (Figure 3E).

Further, to understand the reason why strain RL-WG04 failed to utilize phenol for growth, genes and gene clusters potentially involved in the metabolism of phenol were preliminarily elucidated via whole-genome analysis. RAST annotation indicated that 53 genes were involved in the metabolism of aromatic compounds, including the metabolic pathway of quinate, catechol, benzoate and gentisate (Figure S4). Subsequently, phenol-degrading related pathways and genes were manually checked, and the results indicated that the deficiency of phenol 2-monooxygenase (EC 1.14.13.244) might be the major reason for the failure of phenol degradation (Figure S5).

### 3.4. Synergistic Degradation Analysis of RL-WG04 and RL-LY03

**Figure 4:**
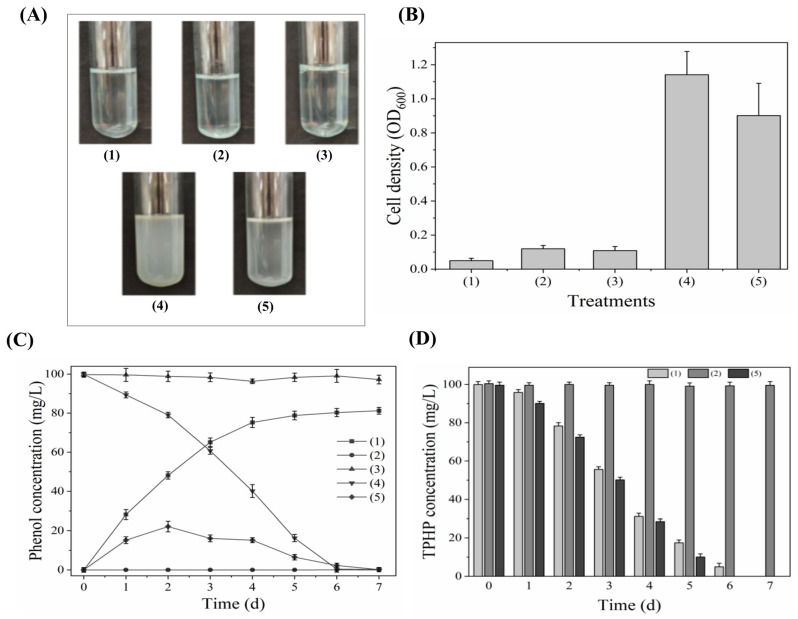
Co-culture of strain RL-WG04 and RL-LY03 for the synergistic degradation of TPHP. (**A**) The cell growth of five treatments; (**B**) the cell density (OD_600_) of five treatments after 7d incubation; (**C**) the generation or degradation of phenol of five treatments; (**D**) the degradation of TPHP in three treatments. The treatments of each graph are associated with Table 1. When TPHP was used as the sole carbon source, the TPHP-degrading bacterium RL-WG04 (Treatment 1), the phenol-degrading bacterium RL-LY03 (Treatment 2), and a combination of both strains (Treatment 5) were added. When phenol was used as the sole carbon source, strain RL-WG04 (Treatment 3) and strain RL-LY03 (Treatment 4) were added.

A novel phenol-degrading bacterial strain was isolated and identified as *Pseudomonas* sp., according to the phylogenetic analysis of the 16S rRNA gene (Supplementary Materials Text S1 and Figure S6). The determination of cell density (OD_600_) indicated that, although strain RL-WG04 could transform TPHP into phenol, strain RL-WG04 failed to utilize TPHP and phenol for growth (Figure 4A). As for strain RL-LY03, it used phenol as a sole carbon source for growth, and the cell density (OD_600_) increased to 1.17 (Figure 4B) but failed to utilize TPHP for growth (Figure 4A). As expected, when TPHP was supplied as the sole carbon source, the simultaneous inoculation of strains RL-WG04 and RL-LY03 achieved an OD_600_ value of 0.92 after 7 d. The growth curve was fitted using the sigmoidal Richards model, yielding a high correlation coefficient (R^2^ = 0.99954) and a growth rate parameter of 0.61 day^−1^ (Figure S7). Additionally, the accumulation or degradation of phenol was analyzed. During the degradation of TPHP by strain RL-WG04, TPHP was completely degraded on the 7th day, while phenol was accumulated in the culture with a final concentration of 81.3 mg/L (~ 8.63 × 10^−4^ mol/L) (Figure 4C), which is approximately three times the initial TPHP concentration (~2.91 × 10^−4^ mol/L). Meanwhile, since strain RL-WG04 could not degrade phenol, the concentration of phenol did not change significantly when phenol was supplemented as the sole carbon source (Figure 4C). Since strain RL-LY03 failed to degrade TPHP (Figure 4D), no accumulation of phenol was observed (Figure 4C). However, strain RL-LY03 could rapidly degrade phenol and completely degrade 100 mg/L of phenol in 7 d. During the synergistic degradation of TPHP by strains RL-WG04 and RL-LY03, TPHP was completely degraded on the 6th day, while the concentration of phenol increased slowly, and the maximum concentration of phenol (26.50 mg/L) appeared on the 3rd day. Subsequently, the concentration of phenol decreased slowly and could not be detected on the 7th day.

### 3.5. Bioremediation of TPHP-Contaminated Sediments

**Figure 5:**
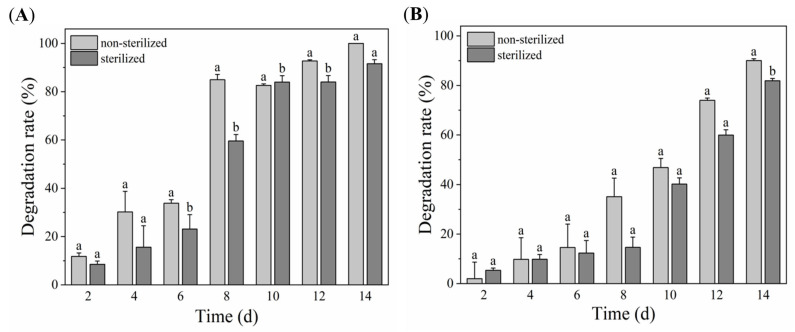
Bioremediation of TPHP-contaminated clay (**A**) and sandy (**B**) sediments by strain RL-WG04. Different lowercase letters indicate statistically significant differences between groups (*p* < 0.05).

As expected, strain RL-WG04 demonstrated effective bioremediation capability for TPHP-contaminated sediments. As illustrated in Figure 5, a lag phase (0 d to 6 d) appeared with the degradation percentages of TPHP in all treatments below 40%. Additionally, the degradation percentages of TPHP in the unsterilized sediments were always higher than the corresponding ones in the sterilized sediments. The maximum degradation percentage of TPHP (100%) on the 14th day was observed in the unsterilized clay sediment, while the one in sandy sediment was 90.04% (the unsterilized sandy sediment).

## 4. Discussion

The isolation of pollutant-degrading bacteria from the environment is a key aspect of biodegradation research. It is of great significance to investigate the role of degrading bacteria in the transformation of environmental pollutants and the bioremediation of contaminated sites. With the large amount of plastic and electronic waste discharged into the environment, OPFR pollution is becoming increasingly serious [15]. As a typical representative of organophosphate esters, TPHP has been widely detected in various environmental matrices, including air [26,27], seawater [28], sediment [27], and biota [29,30,31], while also exhibiting a certain degree of environmental persistence [60]. Standard biodegradability tests based on OECD Test Guideline 301C indicate that TPHP is readily biodegradable, achieving a degradation rate of 83–94% for 100 mg/L TPHP within 28 days (https://www.sigmaaldrich.cn/CN/en/product/aldrich/241288, accessed on 3 January 2026). However, given its widespread environmental occurrence and persistence, research into its biodegradation and bioremediation mechanisms remains of significant environmental importance. Mangrove forests are distributed in tropical and subtropical coastal wetlands, located at the interface of land and sea [61]. These ecosystems have long been impacted by both land-based and marine pollutants due to their interactions with intertidal tidal flats and water environments [62]. Mangrove soil is formed from marine alluvium that has been transported and deposited by rivers and oceans as sediments [63]. Mangrove sediments are characterized by salinization, hypoxia, acidity, and waterlogging [64]. Therefore, mangrove sediments contain diverse and adaptable microbial communities, making them ideal environments for screening efficient pollutant-degrading microorganisms. Thus, mangrove sediment from an intertidal area was selected as the sample, and a TPHP-degrading strain was obtained through enrichment and domestication using TPHP as the sole carbon source. Based on colony morphology, physiological and biochemical characteristics, phylogenetic analysis, and ANI analysis, the strain was identified as *Pseudomonas abyssi* RL-WG04. At present, there are few studies on the biodegradation of TPHP. Most existing degrading strains originate from wastewater treatment plants [65], landfill soil [48,66], and contaminated soil [50]. Few TPHP-degrading bacteria have been isolated from the marine environment. The marine environment is characterized by extreme conditions, such as large temperature variations, high salinity, and slight alkalinity, which make it difficult to directly apply terrestrial degrading bacteria to marine bioremediation. Furthermore, to the best of our knowledge, there has been no report on the degradation of TPHP by *Pseudomonas*. Strain RL-WG04 isolated in this study is the first report of the genus *Pseudomonas* with the ability to degrade TPHP. Therefore, investigating the degradation characteristics and related mechanisms of TPHP by strain RL-WG04 would be helpful for elucidating the fate of TPHP in the marine environment.

The analysis of microbial degradation characteristics represents a significant area of research at the intersection of environmental science and biotechnology. With the growing severity of global environmental pollution, microbial degradation has attracted extensive attention as a green and sustainable approach for pollution control. An in-depth understanding of the effects of different environmental conditions on microbial degradation of pollutants is critical for the development of efficient and stable bioremediation technologies. In this study, the degradation characteristics of strain RL-WG04 demonstrated that it possesses strong adaptability to environmental factors and a broad tolerance range. When the ambient temperature was 30 °C, the pH was 7.0, and the salinity was 2%, the strain achieved the highest degradation rate, degrading 95.22% of TPHP (100 mg/L) by the 5th day. Although the optimal degradation conditions of strain RL-WG04 were characterized as relatively mild, it exhibited excellent alkali resistance, salt tolerance, and degradation efficiency compared to most reported TPHP-degrading bacteria isolated from terrestrial environments. Moreover, it demonstrated strong degradation capability across a wide temperature range. In other words, strain RL-WG04 possessed unique adaptive advantages under conditions approximating those of marine environments. Similar to most degrading bacteria, strain RL-WG04 preferred neutral to alkaline environments [51,52]. However, it could efficiently degrade TPHP at pH 7–9 (degradation rate > 75%), which is highly consistent with the weakly alkaline conditions of seawater. In terms of salinity adaptability, strain RL-WG04 exhibited outstanding salt tolerance, maintaining an effective degradation capacity (degradation rate > 70%) even at a salinity of 6% (*w*/*v*). This range fully covers the actual salinity levels of the ocean and suggests its potential to cope with high salinity fluctuations. In contrast, most TPHP-degrading bacteria reported to date exhibit a narrow salinity tolerance range and cannot efficiently degrade TPHP under high-salt conditions [49,50]. In terms of organic solvent and metal ion tolerance, four organic solvents (*p*-xylene, toluene, biphenyl, and ethyl acetate) showed limited inhibitory effects on the degradation of TPHP by strain RL-WG04. This property may hold promise for applications in industrial wastewater treatment. In addition, seven metal ions (Mn^2+^, Fe^3+^, Ca^2+^, Cu^2+^, Mg^2+^, Zn^2+^ and Co^2+^) inhibited the degradation activity of the strain. Among these, Ca^2+^ and Mg^2+^ reduced the TPHP degradation rate from 97.51% to approximately 75% at a concentration of 1 mM. Strain RL-WG04 was isolated from a marine environment, which typically features high salinity, large temperature fluctuations, and high concentrations of heavy metals and organic pollutants [67]. In this high-stress habitat, microorganisms often develop complex stress responses and detoxification mechanisms for survival.

In-depth analysis of the degradation mechanisms of microbial strains is crucial for advancing our understanding of the fate of pollutants in nature. Elucidating the microbial transformation of pollutants at the molecular level is essential for the design of accurate, efficient, and stable bioremediation systems. Potential metabolic intermediates produced by strain RL-WG04 during TPHP degradation were identified via UHPLC-MS/MS analysis. The biotransformation of TPHP typically involves its conversion to phenol, followed by further utilization of phenol. Most studies have indicated that phenol is a key intermediate in TPHP degradation [51,53,66]. The accumulation of phenol may not only inhibit microbial activity but also cause secondary pollution [68]. Therefore, whether it can be completely degraded is the key to evaluating the degradation efficiency. Although many known TPHP-degrading bacteria can convert TPHP into phenol, these strains often cannot utilize phenol as a growth substrate due to incomplete or entirely absent phenol metabolic pathways [69]. These strains exhibit notable limitations in practical bioremediation applications, making complete pollutant mineralization challenging to achieve. In this study, the degradation capability of the strain was assessed over different time intervals. By the 5th day, the degradation percentage had reached 95.22%, yet no significant bacterial growth was detected, suggesting an inability to fully mineralize and utilize TPHP. Diphenyl phosphate, mono-phenyl phosphate, and phenol were detected during the degradation of TPHP, while phenol was accumulated in the culture. Due to the absence of phenol carboxylase (EC 1.14.13.244), strain RL-WG04 was unable to further metabolize phenol, resulting in an interrupted metabolic pathway. As the culture progressed, TPHP in the system was continuously degraded, while phenol accumulated steadily. Because phenol exerts cytotoxic effects, it inhibits the growth of the strain [70,71]. This inhibition prevents the strain from proliferating normally, ultimately leading to a nearly stagnant growth of strain RL-WG04 throughout the degradation cycle (see Section 3.4, Figure 2B). Therefore, strain RL-WG04 was co-cultured with the phenol-degrading bacterium *Pseudomonas* sp. RL-LY03. The latter strain is capable of utilizing phenol as a carbon source. During synergistic degradation, RL-LY03 assisted RL-WG04 in shortening the lag phase and enabled it to adapt more rapidly to environmental changes, thereby enhancing the degradation efficiency of TPHP. Throughout the cooperative degradation process, the phenol concentration initially increased and subsequently decreased. In contrast, when RL-WG04 was cultured alone, the phenol concentration continued to accumulate. These results indicate that RL-WG04 is unable to decompose or utilize phenol; however, when co-cultured with RL-LY03, the two strains achieved complete degradation of TPHP through a synergistic interaction. This synergistic metabolism provides an effective strategy to achieve complete degradation of complex pollutants, highlighting the importance of functional complementarity within microbial communities in environmental remediation.

In bioremediation, two main strategies are commonly employed: the use of natural microbial communities and artificially constructed co-culture systems. The remediation capacity of natural microbial communities has been validated in numerous practical applications [72,73,74]. The primary advantage of this strategy, which relies on indigenous microorganisms, lies in its ecological adaptability and stability [75]. Because these microorganisms are adapted to the local environment, they pose no risk of ecological invasion. However, remediation processes that depend solely on natural communities are often constrained by low metabolic efficiency [76]. Particularly for recalcitrant exogenous pollutants such as polychlorinated biphenyls and synthetic pesticides, indigenous communities may lack key degrading functional bacteria, leading to prolonged or even stagnant remediation cycles [77]. In contrast, the bottleneck of slow degradation by indigenous microorganisms can be overcome by screening strains with diverse metabolic functions and constructing efficient degrading consortia [76,78,79]. Nevertheless, the limitations of this approach must be considered. Mangrove wetlands, as unique land–sea interface areas, serve as a prime example. In these environments, pollutants are continuously introduced through rivers, tides, and surface runoff. Consequently, they accumulate in sediments, posing a significant threat to ecosystems. This situation creates an urgent practical need for the introduction of effective microbial remediation technologies [80]. However, when introduced into actual polluted environments characterized by complex and dynamic compositions, bacterial strains that exhibit high degradation efficiency under idealized laboratory conditions often fail to replicate their expected performance [81]. Therefore, given the strong adaptability of local microorganisms to specific environmental conditions, bioremediation using indigenous microorganisms directly isolated from the target polluted environment is considered a highly appropriate and efficient strategy [82]. These strains have evolved adaptive mechanisms to tolerate multiple environmental stresses, including high salinity, extreme pH, temperature fluctuations, and elevated pollutant concentrations, in their native habitats. The TPHP-degrading bacterial strain RL-WG04, isolated from the marine environment, demonstrated high potential for remediating TPHP-contaminated mangrove sediments. Within 14 days, it degraded 100% of TPHP (25 mg/kg) in clay mangrove sediment and 90.04% in sandy mangrove sediment. Among them, the clay soil was close to neutral, while the sandy soil was weakly alkaline. The content of organic matter, total nitrogen, and electrical conductivity was higher in clay soil than in sandy soil, indicating higher soil fertility. Throughout the degradation period, the TPHP degradation rate by strain RL-WG04 was consistently higher in clay soil than in sandy soil. These results demonstrate that the strain exhibits better environmental adaptability and a stronger capacity for efficient TPHP degradation in clay soil, which possesses higher fertility and more suitable physicochemical properties. This efficient degradation capability highlights its significance in marine environmental restoration and suggests promising future applications in treating marine pollution. RL-WG04 not only offers a valuable resource for addressing local pollution but also opens new pathways for global environmental restoration practices.

## 5. Conclusions

It is important to study the transformation of environmental pollutants by bacterial degradation and the bioremediation of contaminated areas. In this study, TPHP, a typical aromatic organophosphorus flame retardant, was selected as the target substrate, and a novel TPHP-degrading marine bacterial strain, RL-WG04, was isolated from mangrove sediments. Strain RL-WG04 was identified as *Pseudomonas abyssi,* and its characteristics for TPHP degradation were systematically investigated. The results demonstrate that RL-WG04 exhibits good tolerance to temperatures of 20–50 °C, weakly alkaline conditions (pH 7–9), salinities of 2–6% (*w*/*v*), and organic solvents (*p*-xylene, biphenyl, toluene, and ethyl acetate) at a concentration of 0.5% (*v*/*v*), indicating its strong adaptability to diverse environmental conditions. Kinetic analysis of TPHP degradation by RL-WG04 under optimized conditions revealed that the process aligns well with an improved Gompertz model (R^2^ = 0.997). HPLC-MS identified intermediate metabolites, and the proposed metabolic pathway suggested that RL-WG04 can transform TPHP into phenol via diphenyl phosphate and mono-phenyl phosphate. However, due to the deficiency of phenol 2-monooxygenase, strain RL-WG04 failed to utilize phenol for cell growth. To address this limitation, the phenol-degrading strain *Pseudomonas* sp. RL-LY03 was co-cultured with RL-WG04. The dual-strain co-culture system reduced the lag phase of RL-WG04 and achieved complete TPHP degradation. In terms of practical application, RL-WG04 demonstrated efficient TPHP degradation in bioremediation experiments involving artificially contaminated clay and sandy sediment. This work provides new insights into the fate of TPHP in the marine ecosystem and promotes the bioremediation of TPHP-contaminated environments.

## Figures and Tables

**Table 1:** The experiment design for synergistic degradation of TPHP.

Items	Treatments				
	(1)	(2)	(3)	(4)	(5)
TPHP	●	●	○	○	●
Phenol	○	○	●	●	○
TPHP-degrading strain	●	○	●	○	●
Phenol-degrading strain	○	●	○	●	●

●: means the substrate was supplemented as the sole carbon source; ○: means the substrate was not supplemented as the sole carbon source; ●: means the target strain was inoculated into the treatment; ○: means the target strain was not inoculated into the treatment.

**Table 2:** ANI values between strain RL-WG04 and its closest type strains.

Strain	Accessing Number *	Length (bp)	GC Content (%)	ANI Value (%)
Strain RL-WG04	PRJNA1311828	4,602,770	61.36	/
*Pseudomonas abyssi* MT5^T^	PRJNA406957	4,322,744	61.24	98.38
*Halopseudomonas pachastrellae* JCM 12285^T^	PRJNA323010	3,934,694	61.20	89.88
*Halopseudomonas aestusnigri* CECT 8317^T^	PRJNA224116	3,834,943	60.92	82.80
*Halopseudomonas oceani* CGMCC 1.15195^T^	PRJNA224116	4,167,679	59.92	82.42
*Halopseudomonas salegens* CECT 8338^T^	PRJNA323043	3,796,105	57.69	74.18

*: GenBank accession numbers.

## Data Availability

The original contributions presented in this study are included in the article/Supplementary Materials. Further inquiries can be directed to the corresponding authors.

## Supplementary Materials

The following supporting information can be downloaded at: https://www.mdpi.com/article/10.3390/toxics14040280/s1, Text S1: Isolation of phenol-degrading bacteria; Table S1: Soil characteristics of clay and sandy soil.; Figure S1: Linear fitting between TPHP standard concentration and peak area, A: 20–100 mg/L, B: 4–20 mg/L; Figure S2: Inhibitory effect of TPHP on the growth of the strain RL-WG04; Figure S3: Time-dependent HPLC profiles of TPHP and phenol during degradation (0 d and 5 d); Figure S4: Annotation results of strain RL-WG04 based on RAST system; Figure S5: Benzoic acid metabolism pathway of RL-WG04 in KEGG (the pink part is the hydrolase detected in RL-WG04, and the purple part is the undetected hydrolase); Figure S6: 16S rRNA phylogenetic tree of RL-LY03; Figure S7: The growth kinetic of the co-culture of strains RL-WG04 and RL-LY03 fitted with the Sigmoidal Richards model.
