# Supplementary material for: Biodegradation of Triphenyl Phosphate by a Novel Marine Bacterium Pseudomonas abyssi RL-WG04: Characterization, Metabolic Pathway, Bioremediation and Synergistic Metabolism
Source: Toxics. 2026 Mar 26;14(4):280. doi: 10.3390/toxics14040280 (PMC13120254; doi:10.3390/toxics14040280)
Supplement: Supplementary file 1 [file toxics-14-00280-s001.zip › toxics-4161985-supplementary.pdf]

Supplementary Materials

Biodegradation of triphenyl phosphate by a novel marine bacterium *Pseudomonas abyssi* RL-WG04: Characterization, metabolic pathway, bioremediation and synergistic metabolism

Min Shi[a], Danting Xu[a], John L. Zhou[c], Yang Jia[d], Hanqiao Hu[a, b], Xingyu Jiang[a, b, *], Yanyan Wang[a, *]

a College of Coastal Agricultural Sciences, Guangdong Ocean University, Zhanjiang 524088, China

b South China Branch of National Saline-Alkali Tolerant Rice Technology Innovation Center Zhanjiang, Guangdong 524088, China

c Faculty of Science and Engineering, University of Nottingham Ningbo China, Ningbo 315100, China

d College of Life and Environmental Science, Wenzhou University, Wenzhou 325035, China

* Corresponding author:

Xingyu Jiang, South China Branch of National Saline-Alkali Tolerant Rice Technology Innovation Center Zhanjiang, Guangdong, 524088, China, e-mail address: jiangxingyuhu@163.com

Yanyan Wang, College of Coastal Agricultural Sciences, Guangdong Ocean University, Zhanjiang 524088, China, e-mail address: yanyanwang@gdou.edu.cn

Text S1 Isolation of phenol-degrading bacteria.

Phenol-degrading bacteria were enriched and domesticated using the sole carbon source method. The specific steps are as follows: (a) 1 g of mangrove sediment was inoculated into 100 mL of mineral salt medium (MSM) supplemented with 100 mg/L phenol and incubated in a shaker at 30°C and 180 rpm in the dark for 7 d to enrich phenol-degrading bacteria; (b) 10 mL of the culture was transferred into 90 mL of fresh MSM containing 200 mg/L phenol and cultured under the same conditions for another 7 d; (c) the transfer process was repeated with stepwise increases in phenol concentration up to 500 mg/L; (d) the enriched culture was streaked onto a solid MSM plates containing 100 mg/L phenol and incubated at 30°C in the dark for 3 d; (e) single colonies were picked and inoculated into 10 mL of liquid MSM with 100 mg/L phenol, followed by incubation at 30°C and 180 rpm for 5 d; (f) cultures showing robust growth were filtered through a 0.22 μm membrane, and the phenol degradation rate was measured via HPLC; isolates with degradation rates exceeding 90% were selected; (g) the selected strains were further purified by repeated streaking on solid MSM with 100 mg/L phenol. Steps (e) to (g) were reiterated until uniform colony morphology, stable growth, and consistent degradation rates above 90% were achieved. Finally, the purified strain was streaked on LB agar and incubated at 30°C for 24 h to confirm purity and morphological consistency.

Table S1 Soil characteristics of clay and sandy soil.

Soil type	Collection places	pH	Organic matter (g/kg)	Electric conductivity (μS/cm)	Total nitrogen (g/kg)	TPHP (mg/L)
clay soil	21°147′942″N, 110°442′261″E	7.31	29.44	3,474	0.86	0
sandy soil	21°147′942″N, 110°442′261″E	8.01	7.61	2,596	0.30	0

（A）

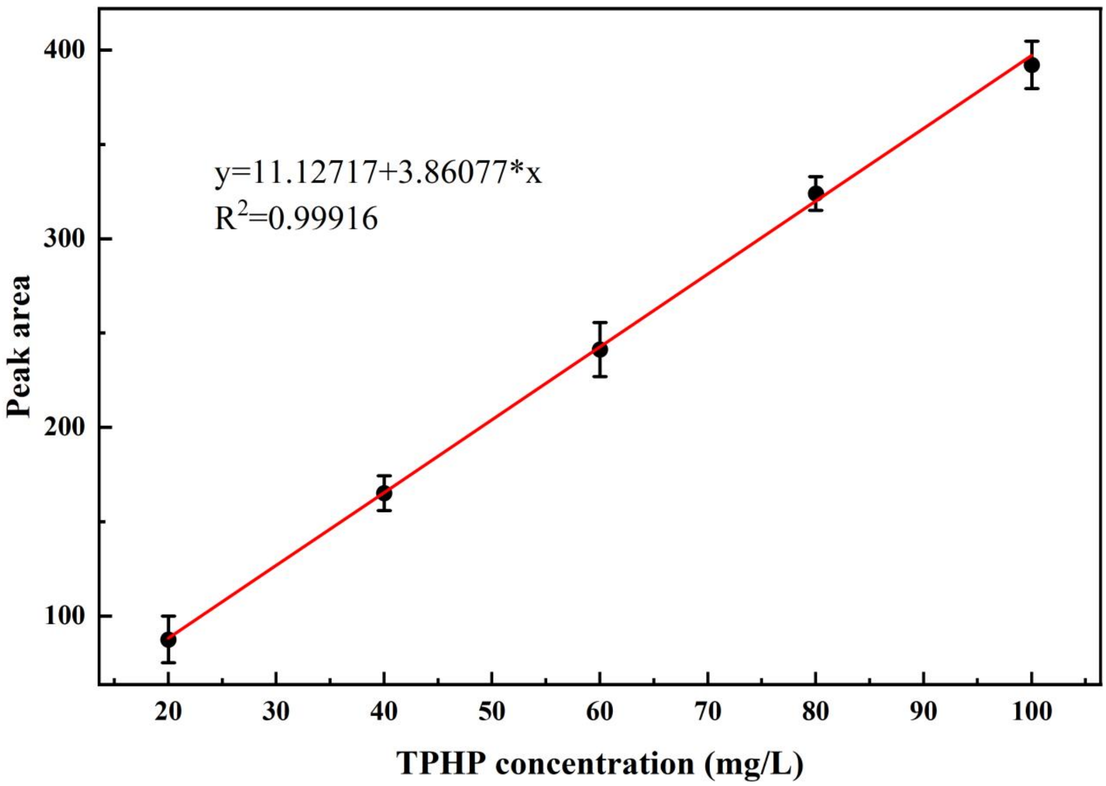

（B）

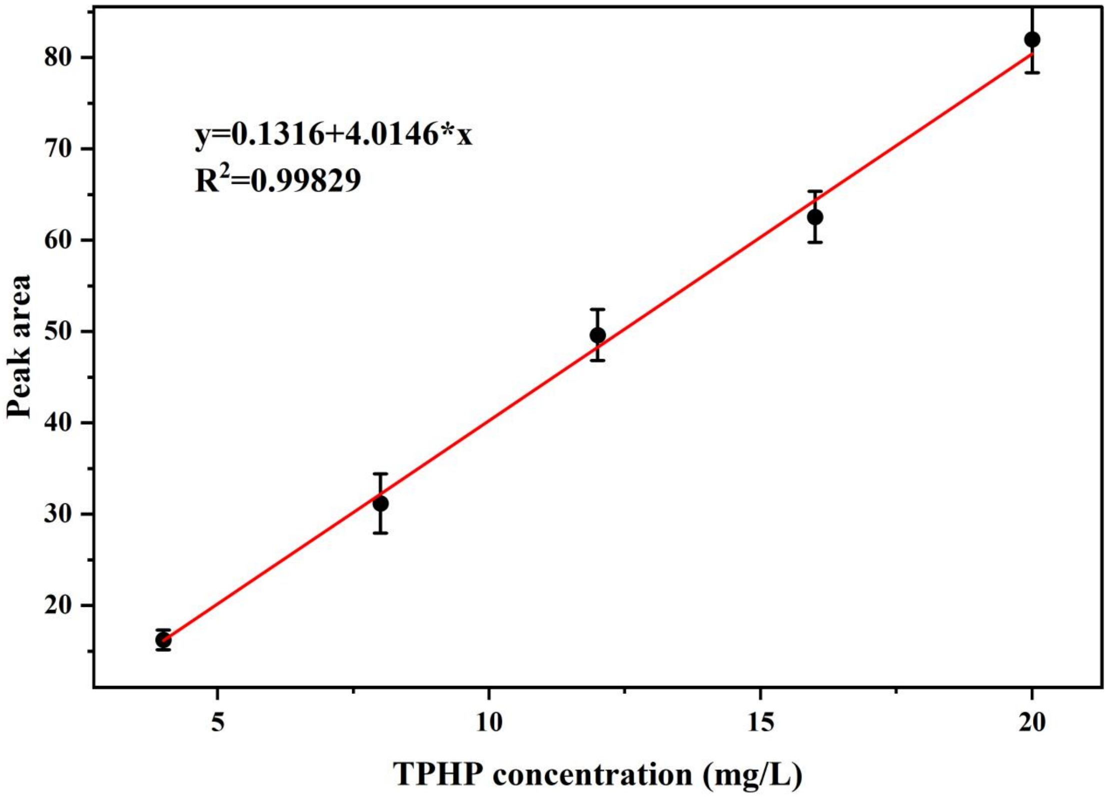

Figure S1 Linear fitting between TPHP standard concentration and peak area

A：20-100 mg/L，B：4-20 mg/L

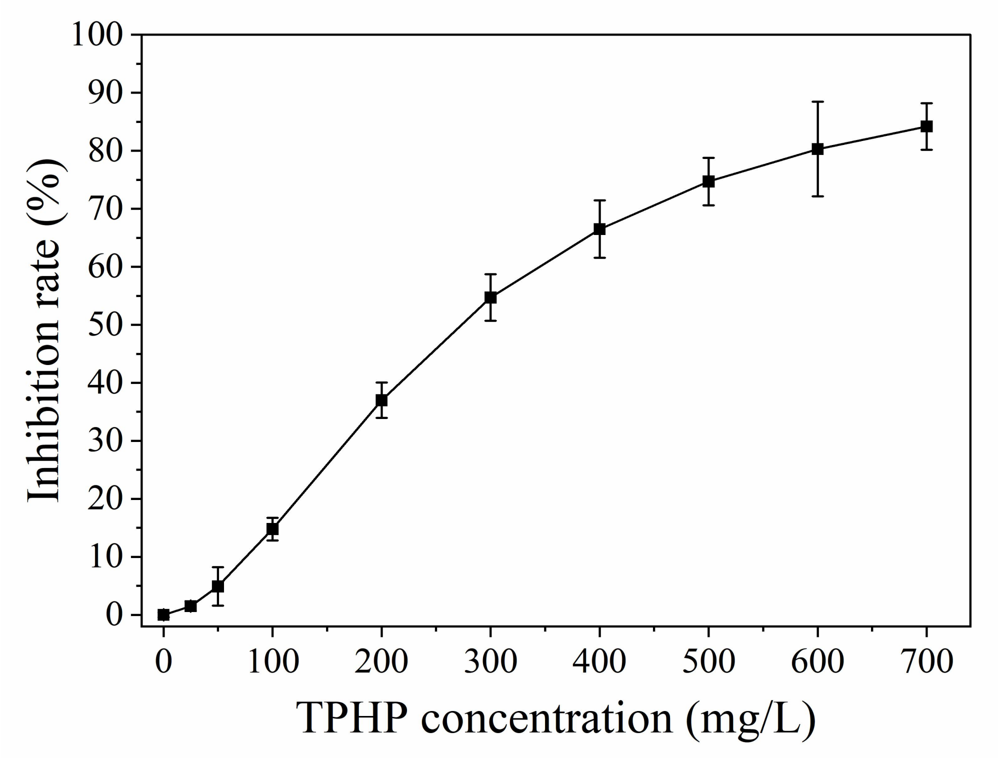

Figure S2 Inhibitory effect of TPHP on the growth of the strain RL-WG04

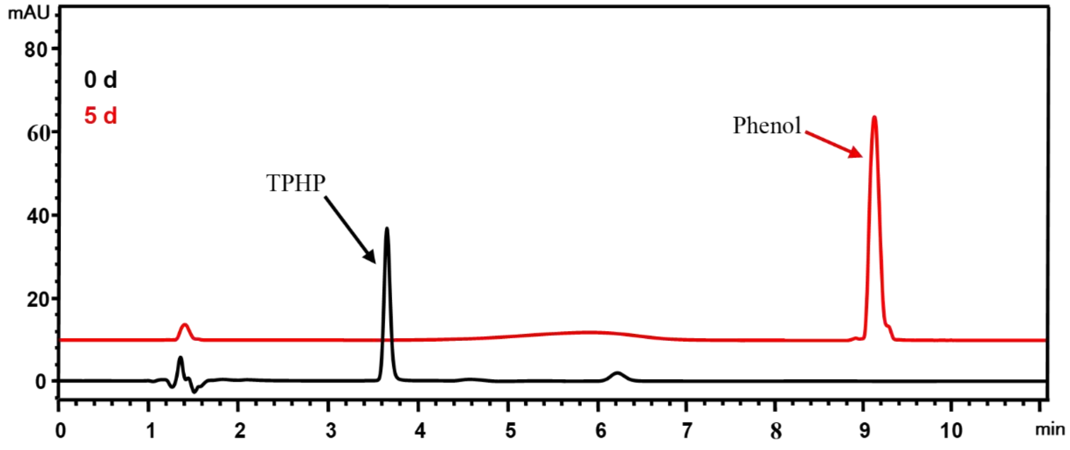

Figure S3 Time-dependent HPLC profiles of TPHP and phenol during degradation (0 d and 5 d)

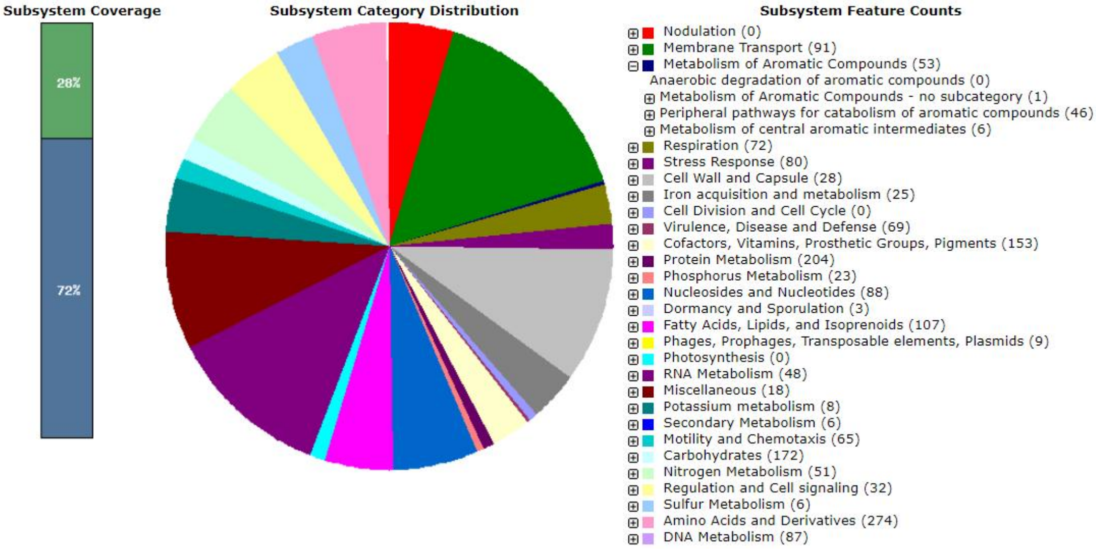

Figure S4 Annotation results of strain RL-WG04 based on RAST system

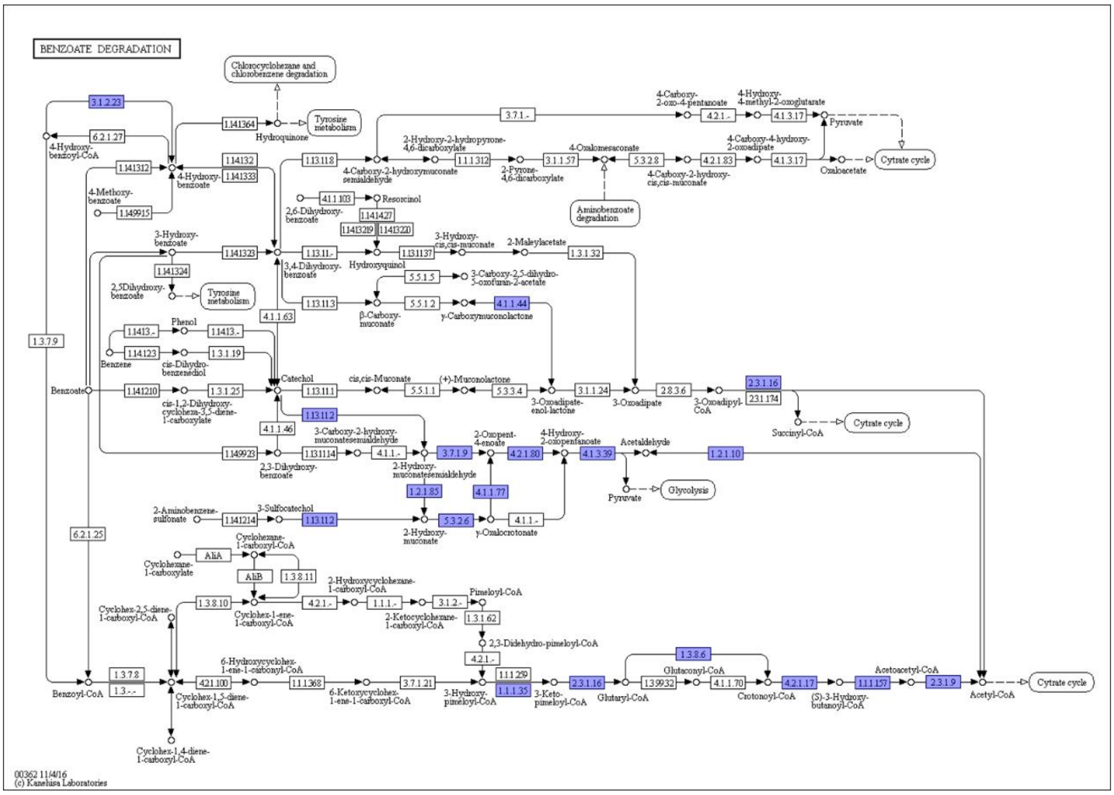

Figure S5 Benzoic acid metabolism pathway of RL-WG04 in KEGG (the pink part is the hydrolase detected in RL-WG04, and the purple part is the undetected hydrolase)

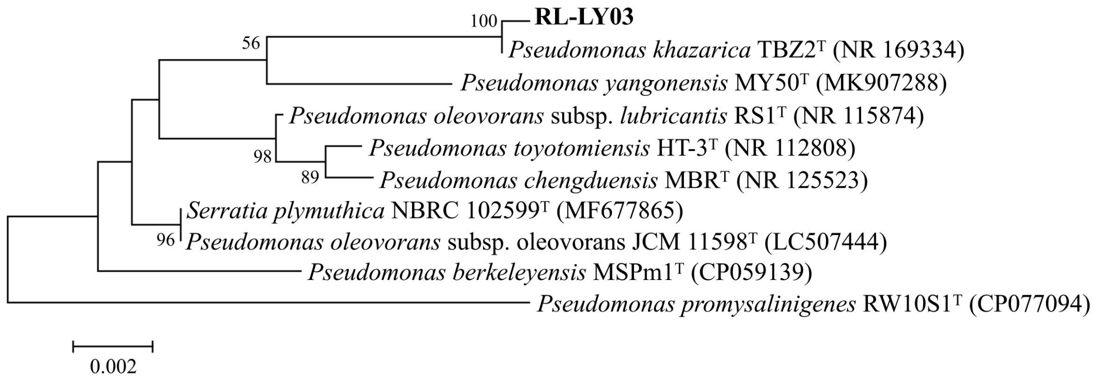

Figure S6 16S rRNA phylogenetic tree of RL-LY03

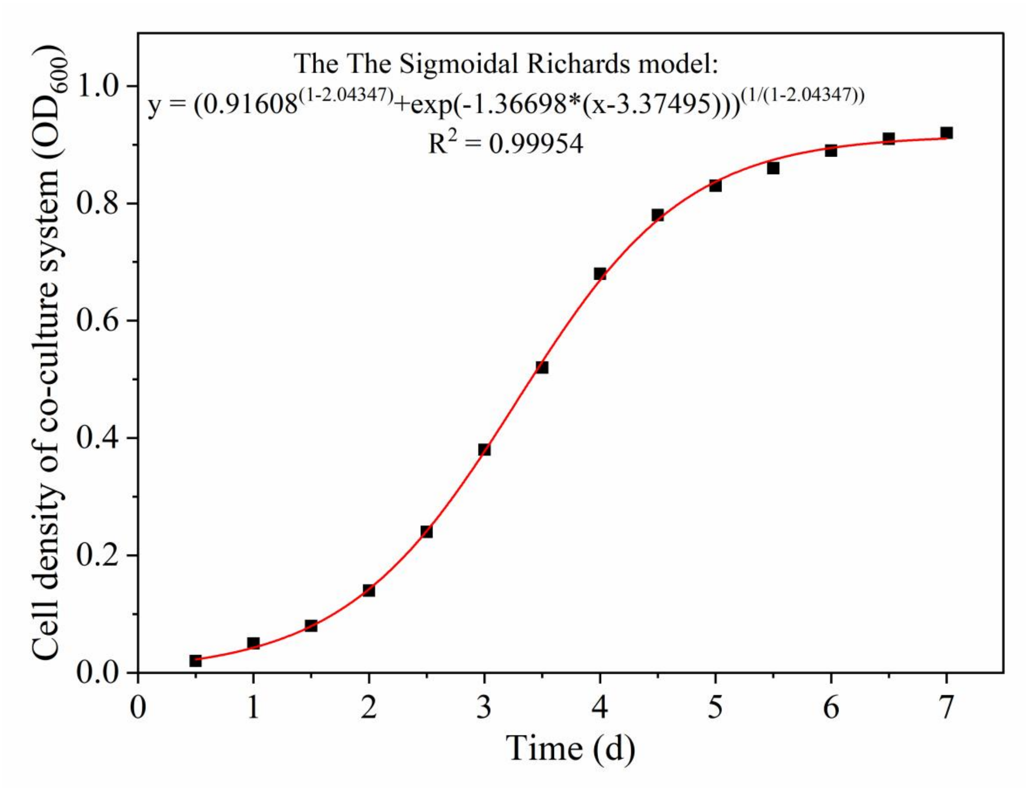

Figure S7 The growth kinetic of the co-culture of strains RL-WG04 and RL-LY03 fitted with the Sigmoidal Richards model
